# 3-(5-Nitro-2-fur­yl)-1-phenyl­prop-2-yn-1-one

**DOI:** 10.1107/S1600536810043850

**Published:** 2010-10-31

**Authors:** Hoong-Kun Fun, Ching Kheng Quah, Nithinchandra, Balakrishna Kalluraya

**Affiliations:** a X-ray Crystallography Unit, School of Physics, Universiti Sains Malaysia, 11800 USM, Penang, Malaysia; b Department of Studies in Chemistry, Mangalore University, Mangalagangotri, Mangalore 574 199, India

## Abstract

In the title compound, C_13_H_7_NO_4_, the 2-furyl ring is essentially planar, with a maximum deviation of 0.004 (1) Å. It is inclined at an angle of 11.69 (4)° to the benzene ring. The nitro group is slightly twisted out of the plane of the 2-furyl ring, with a dihedral angle of 5.72 (8)°. There is a short O⋯C contact of 2.8562 (8) Å (symmetry code: −*x*, −*y*, 2 − *z*). In the crystal packing, mol­ecules are linked *via* a pair of inter­molecular C—H⋯O hydrogen bonds, giving rise to an *R*_2_^2^(10) ring motif. Mol­ecules are further linked into two-dimensional networks parallel to [100] *via* other inter­molecular C—H⋯O hydrogen bonds. The crystal structure is consolidated by C—H⋯π inter­actions.

## Related literature

For general background to the biological activity of nitro­furans, see: Holla *et al.* (1986 ▶, 1987 ▶, 1992 ▶). For the preparation of the title compound, see: Rai *et al.* (2008 ▶). For bond-length data, see: Allen *et al.* (1987 ▶). For hydrogen-bond motifs, see: Bernstein *et al.* (1995 ▶). For the stability of the temperature controller used for the data collection, see: Cosier & Glazer (1986 ▶).
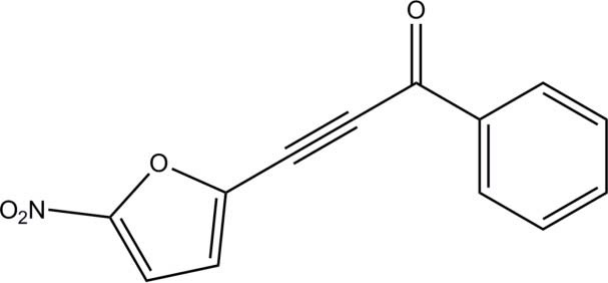


## Experimental

### 

#### Crystal data

C_13_H_7_NO_4_
*M* *_r_* = 241.20
Monoclinic, 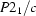

*a* = 10.4685 (2) Å
*b* = 7.3006 (1) Å
*c* = 15.2642 (2) Å
β = 110.867 (1)°
*V* = 1090.07 (3) Å^3^
*Z* = 4
Mo *K*α radiation
μ = 0.11 mm^−1^
*T* = 100 K
0.47 × 0.38 × 0.28 mm

#### Data collection

Bruker SMART APEXII CCD area-detector diffractometer
Absorption correction: multi-scan (*SADABS*; Bruker, 2009 ▶) *T*_min_ = 0.950, *T*_max_ = 0.970
40673 measured reflections
5796 independent reflections
4934 reflections with *I* > 2σ(*I*)
*R*_int_ = 0.030

#### Refinement

*R*[*F* ^2^ > 2σ(*F* ^2^)] = 0.040
*wR*(*F* ^2^) = 0.119
*S* = 1.04
5796 reflections
163 parameters
H-atom parameters constrained
Δρ_max_ = 0.59 e Å^−3^
Δρ_min_ = −0.33 e Å^−3^

### 

Data collection: *APEX2* (Bruker, 2009 ▶); cell refinement: *SAINT* (Bruker, 2009 ▶); data reduction: *SAINT*; program(s) used to solve structure: *SHELXTL* (Sheldrick, 2008 ▶); program(s) used to refine structure: *SHELXTL*; molecular graphics: *SHELXTL*; software used to prepare material for publication: *SHELXTL* and *PLATON* (Spek, 2009 ▶).

## Supplementary Material

Crystal structure: contains datablocks global, I. DOI: 10.1107/S1600536810043850/fj2357sup1.cif

Structure factors: contains datablocks I. DOI: 10.1107/S1600536810043850/fj2357Isup2.hkl

Additional supplementary materials:  crystallographic information; 3D view; checkCIF report

## Figures and Tables

**Table 1:** Hydrogen-bond geometry (Å, °) *Cg*1 is the centroid of the phenyl (C8–C12) ring.

*D*—H⋯*A*	*D*—H	H⋯*A*	*D*⋯*A*	*D*—H⋯*A*
C2—H2⋯O2^i^	0.93	2.44	3.3548 (9)	170
C11—H11⋯O4^ii^	0.93	2.40	3.2487 (9)	152
C3—H3⋯*Cg*1^iii^	0.93	2.86	3.6284 (7)	141

Symmetry codes: (i) 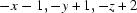; (ii) 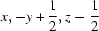; (iii) 
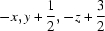
.

**Fig. 1.**
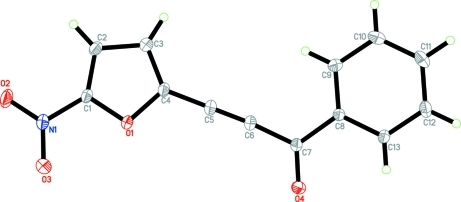
The molecular structure of the title compound showing 50% probability displacement ellipsoids for non-H atoms and the atom-numbering scheme.

**Fig. 2.**
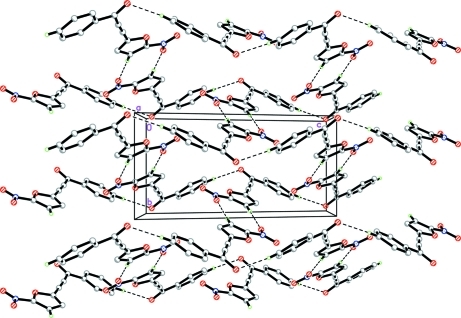
The crystal structure of the title compound, viewed along the a axis. H atoms not involved in hydrogen bonds (dashed lines) have been omitted for clarity.

## supplementary crystallographic information

### Comment

Nitrofurans are class of synthetic compounds characterized by the presence of 5-nitro-2-furyl group. The presence of nitro group in the position-5 of the molecule conferred antibacterial activity (Holla *et al.*, 1986). A number of nitrofurans have attained commercial utility as antibacterial agents in humans and in veterinary medicine because of their broad spectrum of activity (Holla *et al.*, 1992; Holla *et al.*, 1987). 1-Aryl-3-(5-nitro-2-furyl)-2-propyn-1-ones were prepared by the hydrobromination of 2,3-dibromo-1-aryl-3-(5-nitro-2-furyl)-2-propan-1-ones in the presence of triethylamine in benzene medium. The dibromopropanones were in turn obtained by the bromination of 1-aryl-3-(5-nitro-2-furyl)-2-propen-1-ones. Acid catalysed condensation of acetophenones with 5- nitrofuraldiacetate in acetic acid yielded the required 1-aryl-3-(5-nitro-2-furyl)-2-propen-1-ones called chalcones (Rai *et al.*, 2008).

In the title molecule (Fig. 1), the 2-furyl (O1/C1–C4) ring is essentially planar (maximum deviation = 0.004 (1) Å for atoms O1) and is inclined at an angle of 11.69 (4)° with the phenyl ring (C11–C16), which indicates they are nearly parallel to each other. The nitro group (N1/O1/O2) is slightly twisted away from the attached 2-furyl ring [dihedral angle = 5.72 (8)]. There is a short O4···C1 contact (symmetry code : -x, -y, 2 - z) with distance = 2.8562 (8) Å which is shorter than the sum of van der Waals radii of the O and C atoms. Bond lengths (Allen *et al.*, 1987) and angles are within normal ranges.

In the crystal packing (Fig. 2), the molecules are linked *via* a pair of intermolecular C2—H2···O2 hydrogen bonds, displaying R_2_^2^(10) ring motifs (Bernstein *et al.*, 1995). The molecules are further linked into two-dimensional networks parallel to (100) *via* intermolecular C11—H11···O4 hydrogen bonds. The crystal structure is further consilidated by C3—H3···Cg1 (Table 1), where Cg1 is the centroid of C8–C13 phenyl ring.

### Experimental

To a stirred solution of 2,3-dibromo-3-(5-nitro-2-furyl)-1-phenylpropan-1-one (0.01 mol) in dry benzene (100 ml), a solution of triethylamine (0.04 mol) in dry benzene (30 ml) was added. The reaction mixture was stirred at room temperature for 24 h. The resulting mass of triethylamine hydrobromide was removed by filtration and the filtrate was concentrated by distilling the benzene under reduced pressure. The concentrated solution was cooled to room temperature. The product formed was collected by filtration and washed with ethanol. It was then recrystallized from ethanol. Crystals suitable for X-ray analysis were obtained from 1:2 mixtures of DMF and ethanol by slow evaporation.

### Refinement

All H atoms were positioned geometrically and refined using a riding model with C—H = 0.93 Å and *U*_iso_(H) = 1.2*U*_eq_(C). The highest residual electron density peak is located at 0.70 Å from C13 and the deepest hole is located at 1.10 Å from C1.

### Crystal data

**Table:**

C_13_H_7_NO_4_	*F*(000) = 496
*M**_r_* = 241.20	*D*_x_ = 1.470 Mg m^−^^3^
Monoclinic, *P*2_1_/*c*	Mo *K*α radiation, λ = 0.71073 Å
Hall symbol: -P 2ybc	Cell parameters from 9876 reflections
*a* = 10.4685 (2) Å	θ = 2.8–37.6°
*b* = 7.3006 (1) Å	µ = 0.11 mm^−^^1^
*c* = 15.2642 (2) Å	*T* = 100 K
β = 110.867 (1)°	Block, brown
*V* = 1090.07 (3) Å^3^	0.47 × 0.38 × 0.28 mm
*Z* = 4	

### Data collection

**Table:**

Bruker SMART APEXII CCD area-detector diffractometer	5796 independent reflections
Radiation source: fine-focus sealed tube	4934 reflections with *I* > 2σ(*I*)
graphite	*R*_int_ = 0.030
φ and ω scans	θ_max_ = 37.7°, θ_min_ = 2.1°
Absorption correction: multi-scan (*SADABS*; Bruker, 2009)	*h* = −17→17
*T*_min_ = 0.950, *T*_max_ = 0.970	*k* = −12→12
40673 measured reflections	*l* = −25→26

### Refinement

**Table:**

Refinement on *F*^2^	Primary atom site location: structure-invariant direct methods
Least-squares matrix: full	Secondary atom site location: difference Fourier map
*R*[*F*^2^ > 2σ(*F*^2^)] = 0.040	Hydrogen site location: inferred from neighbouring sites
*wR*(*F*^2^) = 0.119	H-atom parameters constrained
*S* = 1.04	*w* = 1/[σ^2^(*F*_o_^2^) + (0.0678*P*)^2^ + 0.1893*P*] where *P* = (*F*_o_^2^ + 2*F*_c_^2^)/3
5796 reflections	(Δ/σ)_max_ = 0.002
163 parameters	Δρ_max_ = 0.59 e Å^−^^3^
0 restraints	Δρ_min_ = −0.33 e Å^−^^3^

### Special details

**Table:**

Experimental. The crystal was placed in the cold stream of an Oxford Cyrosystems Cobra open-flow nitrogen cryostat (Cosier & Glazer, 1986) operating at 100.0 (1) K.
Geometry. All esds (except the esd in the dihedral angle between two l.s. planes) are estimated using the full covariance matrix. The cell esds are taken into account individually in the estimation of esds in distances, angles and torsion angles; correlations between esds in cell parameters are only used when they are defined by crystal symmetry. An approximate (isotropic) treatment of cell esds is used for estimating esds involving l.s. planes.
Refinement. Refinement of F^2^ against ALL reflections. The weighted R-factor wR and goodness of fit S are based on F^2^, conventional R-factors R are based on F, with F set to zero for negative F^2^. The threshold expression of F^2^ > 2sigma(F^2^) is used only for calculating R-factors(gt) etc. and is not relevant to the choice of reflections for refinement. R-factors based on F^2^ are statistically about twice as large as those based on F, and R- factors based on ALL data will be even larger.

### Fractional atomic coordinates and isotropic or equivalent isotropic displacement parameters (Å^2^)

**Table:**

	*x*	*y*	*z*	*U*_iso_*/*U*_eq_	
O1	−0.11630 (5)	0.27415 (7)	1.04906 (3)	0.01471 (9)	
O2	−0.39290 (6)	0.35131 (8)	1.11730 (4)	0.02350 (11)	
O3	−0.19217 (6)	0.23310 (8)	1.19322 (4)	0.02173 (10)	
O4	0.31281 (5)	0.03921 (7)	0.98678 (3)	0.01702 (9)	
N1	−0.27662 (6)	0.30540 (8)	1.12394 (4)	0.01630 (10)	
C1	−0.23941 (6)	0.33853 (8)	1.04384 (4)	0.01429 (10)	
C2	−0.31207 (6)	0.41459 (9)	0.95952 (5)	0.01643 (11)	
H2	−0.3984	0.4675	0.9412	0.020*	
C3	−0.22679 (6)	0.39527 (9)	0.90560 (5)	0.01704 (11)	
H3	−0.2463	0.4335	0.8441	0.020*	
C4	−0.11011 (6)	0.30936 (9)	0.96198 (4)	0.01464 (10)	
C5	0.00843 (6)	0.25151 (9)	0.94661 (5)	0.01623 (11)	
C6	0.10953 (6)	0.20010 (9)	0.93282 (4)	0.01633 (11)	
C7	0.23677 (6)	0.13782 (8)	0.92494 (4)	0.01320 (10)	
C8	0.27014 (6)	0.19941 (8)	0.84344 (4)	0.01292 (10)	
C9	0.17614 (7)	0.29769 (9)	0.76987 (4)	0.01676 (11)	
H9	0.0897	0.3240	0.7707	0.020*	
C10	0.21317 (8)	0.35576 (10)	0.69539 (5)	0.02011 (12)	
H10	0.1514	0.4214	0.6463	0.024*	
C11	0.34271 (8)	0.31575 (10)	0.69438 (5)	0.02020 (12)	
H11	0.3673	0.3559	0.6448	0.024*	
C12	0.43585 (7)	0.21588 (10)	0.76724 (5)	0.01871 (11)	
H12	0.5219	0.1886	0.7658	0.022*	
C13	0.39983 (6)	0.15728 (9)	0.84181 (4)	0.01523 (10)	
H13	0.4615	0.0904	0.8904	0.018*	

### Atomic displacement parameters (Å^2^)

**Table:**

	*U*^11^	*U*^22^	*U*^33^	*U*^12^	*U*^13^	*U*^23^
O1	0.01147 (17)	0.01760 (19)	0.01634 (19)	0.00183 (14)	0.00653 (14)	0.00090 (15)
O2	0.0173 (2)	0.0286 (3)	0.0299 (3)	0.00376 (18)	0.0150 (2)	−0.0001 (2)
O3	0.0219 (2)	0.0261 (2)	0.0180 (2)	0.00245 (18)	0.00804 (18)	0.00227 (18)
O4	0.0181 (2)	0.0185 (2)	0.01441 (18)	0.00230 (15)	0.00568 (15)	0.00193 (15)
N1	0.0158 (2)	0.0164 (2)	0.0192 (2)	0.00019 (17)	0.00932 (18)	−0.00110 (17)
C1	0.0117 (2)	0.0154 (2)	0.0176 (2)	0.00109 (17)	0.00733 (18)	−0.00024 (18)
C2	0.0131 (2)	0.0173 (2)	0.0191 (2)	0.00249 (18)	0.00597 (19)	0.00072 (19)
C3	0.0155 (2)	0.0189 (3)	0.0172 (2)	0.00230 (19)	0.00642 (19)	0.00149 (19)
C4	0.0132 (2)	0.0159 (2)	0.0166 (2)	0.00058 (17)	0.00742 (18)	−0.00012 (18)
C5	0.0143 (2)	0.0174 (2)	0.0190 (2)	−0.00023 (18)	0.0085 (2)	−0.00129 (19)
C6	0.0148 (2)	0.0186 (3)	0.0177 (2)	0.00069 (19)	0.00826 (19)	−0.00003 (19)
C7	0.0124 (2)	0.0142 (2)	0.0139 (2)	−0.00032 (16)	0.00575 (17)	−0.00142 (17)
C8	0.0122 (2)	0.0142 (2)	0.0128 (2)	0.00024 (16)	0.00488 (17)	0.00001 (16)
C9	0.0150 (2)	0.0180 (2)	0.0157 (2)	0.00193 (19)	0.00354 (19)	0.00166 (19)
C10	0.0241 (3)	0.0193 (3)	0.0146 (2)	−0.0002 (2)	0.0040 (2)	0.0030 (2)
C11	0.0263 (3)	0.0213 (3)	0.0146 (2)	−0.0062 (2)	0.0092 (2)	−0.0006 (2)
C12	0.0179 (3)	0.0233 (3)	0.0179 (2)	−0.0032 (2)	0.0100 (2)	−0.0016 (2)
C13	0.0126 (2)	0.0190 (2)	0.0150 (2)	0.00056 (18)	0.00601 (18)	0.00020 (18)

### Geometric parameters (Å, °)

**Table:**

O1—C1	1.3472 (7)	C6—C7	1.4525 (8)
O1—C4	1.3779 (8)	C7—C8	1.4770 (8)
O2—N1	1.2315 (7)	C8—C9	1.3990 (8)
O3—N1	1.2298 (8)	C8—C13	1.4010 (8)
O4—C7	1.2278 (8)	C9—C10	1.3915 (9)
N1—C1	1.4298 (8)	C9—H9	0.9300
C1—C2	1.3586 (9)	C10—C11	1.3927 (11)
C2—C3	1.4205 (9)	C10—H10	0.9300
C2—H2	0.9300	C11—C12	1.3953 (10)
C3—C4	1.3701 (9)	C11—H11	0.9300
C3—H3	0.9300	C12—C13	1.3880 (9)
C4—C5	1.4073 (8)	C12—H12	0.9300
C5—C6	1.2100 (8)	C13—H13	0.9300
			
C1—O1—C4	104.51 (5)	C6—C7—C8	118.26 (5)
O3—N1—O2	125.05 (6)	C9—C8—C13	120.49 (5)
O3—N1—C1	118.47 (5)	C9—C8—C7	121.50 (5)
O2—N1—C1	116.48 (6)	C13—C8—C7	118.01 (5)
O1—C1—C2	113.56 (5)	C10—C9—C8	119.41 (6)
O1—C1—N1	115.88 (5)	C10—C9—H9	120.3
C2—C1—N1	130.40 (5)	C8—C9—H9	120.3
C1—C2—C3	104.66 (5)	C9—C10—C11	120.06 (6)
C1—C2—H2	127.7	C9—C10—H10	120.0
C3—C2—H2	127.7	C11—C10—H10	120.0
C4—C3—C2	106.57 (6)	C10—C11—C12	120.49 (6)
C4—C3—H3	126.7	C10—C11—H11	119.8
C2—C3—H3	126.7	C12—C11—H11	119.8
C3—C4—O1	110.71 (5)	C13—C12—C11	119.86 (6)
C3—C4—C5	132.41 (6)	C13—C12—H12	120.1
O1—C4—C5	116.88 (5)	C11—C12—H12	120.1
C6—C5—C4	179.25 (7)	C12—C13—C8	119.67 (6)
C5—C6—C7	175.08 (7)	C12—C13—H13	120.2
O4—C7—C6	118.85 (5)	C8—C13—H13	120.2
O4—C7—C8	122.88 (5)		
			
C4—O1—C1—C2	−0.69 (7)	O4—C7—C8—C9	174.06 (6)
C4—O1—C1—N1	175.17 (5)	C6—C7—C8—C9	−7.19 (9)
O3—N1—C1—O1	4.75 (9)	O4—C7—C8—C13	−6.23 (9)
O2—N1—C1—O1	−174.73 (6)	C6—C7—C8—C13	172.52 (6)
O3—N1—C1—C2	179.76 (7)	C13—C8—C9—C10	−0.90 (10)
O2—N1—C1—C2	0.28 (11)	C7—C8—C9—C10	178.80 (6)
O1—C1—C2—C3	0.46 (8)	C8—C9—C10—C11	0.11 (10)
N1—C1—C2—C3	−174.65 (7)	C9—C10—C11—C12	0.64 (11)
C1—C2—C3—C4	−0.02 (7)	C10—C11—C12—C13	−0.61 (11)
C2—C3—C4—O1	−0.40 (7)	C11—C12—C13—C8	−0.18 (10)
C2—C3—C4—C5	178.65 (7)	C9—C8—C13—C12	0.93 (10)
C1—O1—C4—C3	0.65 (7)	C7—C8—C13—C12	−178.78 (6)
C1—O1—C4—C5	−178.56 (6)		

### Hydrogen-bond geometry (Å, °)

**Table:**

*D*—H···*A*	*D*—H	H···*A*	*D*···*A*	*D*—H···*A*
C2—H2···O2^i^	0.93	2.44	3.3548 (9)	170
C11—H11···O4^ii^	0.93	2.40	3.2487 (9)	152
C3—H3···Cg1^iii^	0.93	2.86	3.6284 (7)	141

Symmetry codes: (i) −*x*−1, −*y*+1, −*z*+2; (ii) *x*, −*y*+1/2, *z*−1/2; (iii) −*x*, *y*+1/2, −*z*+3/2.
